# *Monotropastrum kirishimense* (Ericaceae), a new mycoheterotrophic plant from Japan based on multifaceted evidence

**DOI:** 10.1007/s10265-022-01422-8

**Published:** 2022-11-29

**Authors:** Kenji Suetsugu, Shun K. Hirota, Tian-Chuan Hsu, Shuichi Kurogi, Akio Imamura, Yoshihisa Suyama

**Affiliations:** 1 Department of Biology, Graduate School of Science, Kobe University, Kobe, 657-8501 Japan; 2 The Institute for Advanced Research, Kobe University, 1-1 Rokkodai, Nada-ku, Kobe, 657-8501 Japan; 3 Field Science Center, Graduate School of Agricultural Science, Tohoku University, 232-3 Yomogida, Naruko-Onsen, Osaki, Miyagi 989-6711 Japan; 4 Botanical Garden Division, Taiwan Forestry Research Institute, No. 53, Nanhai Rd., Taipei, 100 Taiwan; 5 Miyazaki Prefectural Museum of Nature and History, 2-4-4, Jingû, Miyazaki 880-0053 Japan; 6 Hokkaido University of Education, Sapporo, 002-8501 Japan

**Keywords:** Fungal association, Integrative taxonomy, Mycoheterotrophy, Mycorrhizas, Reproductive isolation, SNP data, Speciation, Species delimitation

## Abstract

Due to their reduced morphology, non-photosynthetic plants have been one of the most challenging groups to delimit to species level. The mycoheterotrophic genus *Monotropastrum*, with the monotypic species *M. humile*, has been a particularly taxonomically challenging group, owing to its highly reduced vegetative and root morphology. Using integrative species delimitation, we have focused on Japanese *Monotropastrum*, with a special focus on an unknown taxon with rosy pink petals and sepals. We investigated its flowering phenology, morphology, molecular identity, and associated fungi. Detailed morphological investigation has indicated that it can be distinguished from *M. humile* by its rosy pink tepals and sepals that are generally more numerous, elliptic, and constantly appressed to the petals throughout its flowering period, and by its obscure root balls that are unified with the surrounding soil, with root tips that hardly protrude. Based on genome-wide single-nucleotide polymorphisms, molecular data has provided clear genetic differentiation between this unknown taxon and *M. humile*. *Monotropastrum humile* and this taxon are associated with different *Russula* lineages, even when they are sympatric. Based on this multifaceted evidence, we describe this unknown taxon as the new species *M. kirishimense*. Assortative mating resulting from phenological differences has likely contributed to the persistent sympatry between these two species, with distinct mycorrhizal specificity.

**Supplementary Information:**

The online version contains supplementary material available at 10.1007/s10265-022-01422-8.

## Introduction

The subfamily Monotropoideae (Ericaceae), is distributed throughout the Northern Hemisphere (Bidartondo and Bruns 2001; Kron et al. 2002). It is characterized by its achlorophyllous and fully mycoheterotrophic habit, with scale-like leaves, bisexual and actinomorphic flowers, free sepals and petals, and a superior 1–6 locule ovary (Wallace 1987; Wu et al. 2016). As currently circumscribed, Monotropoideae contains ca. 15 species in 12 genera: *Eremotropa* Andres, *Hypopitys* Hill, *Allotropa* Torr. & A.Gray, *Hemitomes* A.Gray, *Monotropa* L., *Monotropastrum* Andres, *Monotropsis* Schwein., *Pityopus* Small, *Pleuricospora* A.Gray, *Pterospora* Nutt., *Cheilotheca* Hook.f., and *Sarcodes* Torr. (Rose and Freudenstein 2014; Wallace 1975; Wu et al. 2016; Zhao et al. 2019). Although its center of species diversity is western North America, with seven endemic species, at least five species, including *Monotropastrum humile* (D.Don) Hara, three *Cheilotheca* species, and *Eremotropa sciaphila* Andres, are Asian endemics (Wallace 1975; Wu et al. 2016; Zhao et al. 2019).

The monotypic genus *Monotropastrum*, including *M. humile*, is widely distributed in East Asia, from the Himalayas to Japan (POWO 2022). However, the taxonomic treatment of *Monotropastrum* is still confusing in terms of varietal recognition and generic attribution (Tsukaya et al. 2008). Although in some cases it has been separated into two varieties, namely *M. humile* var. *humile* and *M. humile* var. *glaberrimum* Hara (Hara 1961, 1965), recognition of *M. humile* var. *glaberrimum* has often been neglected (POWO 2022; Qin and Wallace 2005). Consequently, *M. humile* has often been recognized as having considerable morphological variation (Qin and Wallace 2005).

The generic classification of *Monotropastrum* is also confusing. *Monotropastrum* shares ovary and fruit characteristics with *Cheilotheca*, including unilocular ovaries with parietal placentation and baccate fruits (Andres 1935, 1936). This has led to *Monotropastrum* often being considered synonymous with *Cheilotheca* (Hsu et al. 1998; Keng 1974; Keng and Hsieh 1978). Therefore, Keng and Hsieh (1978) transferred *M. humile* and *M. humile* var. *glaberrimum* to *Cheilotheca humilis* (D.Don) H.Keng and *C. humilis* var. *glaberrimum* (H.Hara) H.Keng & Hsieh, respectively. However, despite their similarities, there are some substantial differences between *Monotropastrum* and *Cheilotheca*, such as the petals lacking or having a thickened apex (Wallace 1987). Considering the genetic distance between *Monotropastrum* and *Cheilotheca* is greater than that between other genera in the Monotropoideae, *Monotropastrum* and *Monotropa* (Tsukaya et al. 2008), we highlight that *Monotropastrum* and *Cheilotheca* should be accepted, with *M. humile* var. *glaberrimum* transferred into *Cheilotheca*, as proposed by Tsukaya et al. (2008).

Regarding the taxonomic treatment of *Monotropastrum*, it is also noteworthy that an unknown *Monotropastrum* taxon, with rosy pink petals and sepals, has long been recognized around Kirishima, Kagoshima Prefecture, Japan (Imamura and Kurogi 2003). It is morphologically similar morphologically to *M. humile* f. *humile* in having nodding flowers at anthesis, petals without a thickened apex, a single-loculed ovary with parietal placentation, and baccate fruits. Consequently, it has tentatively been treated as a color variant of *M. humile*, known as *M. humile* f. *roseum* Honda (Imamura and Kurogi 2003). However, the flowering seasons for this taxon and *M. humile* do not overlap (Kurogi, unpublished data), and their mycorrhizal morphology and root systems differ considerably (Imamura and Kurogi 2003). Therefore, this unknown taxon may be a cryptic species rather than a color variant. Organisms with reduced morphology have always presented a challenge for systematists because of the relative paucity of characters (Barrett and Freudenstein 2011). Non-photosynthetic plants with highly reduced leaves are, therefore, prime candidates for being cryptic species (Barrett and Freudenstein 2011; de Vega et al. 2008; Thorogood et al. 2008). Morphological changes accompanying the shift to mycoheterotrophy include the loss of the leaf laminae, and reduced underground organs. Therefore, it is crucial to examine multiple characteristics for species delimitation in mycoheterotrophic species (Barrett and Freudenstein 2011).

Here, we have applied an integrative taxonomic approach to test whether this taxon should be considered a distinct species from *Monotropastrum humile.* We investigated discontinuities in flowering phenology and floral morphology that could uniquely diagnose this taxon. We then reconstructed the phylogenetic relationships to examine whether the taxon represents distinct evolutionary lineages, based on MIG-seq [multiplexed inter-simple sequence repeat (ISSR) genotyping by sequencing] data. Finally, given that fungal-associate identity may be relevant in delimiting mycoheterotrophic taxa (Barrett and Freudenstein 2011; Barrett et al. 2022; Freudenstein and Barrett 2014), we investigated the mycorrhizal fungal communities of this taxon and *M. humile*, including at a sympatric site, using high-throughput DNA sequencing. Our multifaceted evidence leads us to conclude that this taxon is morphologically, phenologically, phylogenetically, and ecologically distinct, and should, therefore, be recognized as a separate species. Consequently, we have described it as a new species, *M. kirishimense* Suetsugu. Our data are consistent with a study that has shown that fungal host utilization enhances species delimitation in mycoheterotrophic orchids (Freudenstein and Barrett 2014). Our study presents the exciting possibility that a host shift in *M. kirishimense*, toward a specific *Russula* lineage, triggered ecological speciation.

## Materials and methods

### Specimen collection and preservation

We collected 50 *Monotropastrum kirishimense* plants encompassing ten Japanese populations. A total of 38 individuals of *M. humile*, including five *M. humile* f. *roseum* plants, were collected throughout Japan and Taiwan from a total of eight populations, as shown in Table S1. For comparative study, we also collected one specimen from Vietnam [*Hsu 10691* (STG00764), hereafter referred to as *Monotropastrum* sp. 1]. This specimen differs morphologically from typical *M. humile*, and has glabrous flowers and broad, somewhat ridged fruits (Fig. S1). To minimize disturbance to the local populations, the minimum number of samples required for molecular analysis were collected. However, at least one voucher specimen encompassing the entire plant was deposited in KYO, MZ, TAIF, TI and TNS representing each population. Scale leaves for DNA analysis were immediately dried using silica gel and stored at room temperature until DNA extraction, while 1–3 root fragments (ca. 1 mm in diameter and 3–5 mm in length) were collected from each specimen for molecular barcoding of mycorrhizal fungi. Each root sample was transferred to a 1.5 mL tube containing 99.5% ethanol, and stored at − 20 °C. The herbarium acronyms follow Index Herbariorum (Thiers 2021).

### Morphological observation

We compared the morphological characters of *Monotropastrum kirishimense*, *M. humile*, and *Monotropastrum* sp. 1, using the samples listed in Table S1. The morphological variation in *M. kirishimense* and *M. humile* was also investigated by reviewing the literature and herbarium specimens (at KYO, TAIF and TI) from other localities. The morphological characters were visually observed under a stereomicroscope and measured using a digital caliper. We note that *M. kirishimense* is somewhat similar to *M. humile* f. *roseum* described from Sadogashima, Niigata Prefecture, Japan, and has rosy pink flowers. Therefore, we examined the *M. humile* f. *roseum* type specimen at TI (TI00205063) in detail, and additional *M. humile* f. *roseum* specimens from other localities, to identify consistent morphological differences between *M. kirishimense* and *M. humile*.

### Flowering phenology analysis

Field observations of the flowering phenology from a sympatric site were used to determine whether differences in the flowering phenology play a role in maintaining reproductive integrity between *Monotropastrum kirishimense* and *M. humile*. For quantitative comparison, we counted the scapes of *M. kirishimense* and *M. humile* from the Onami population (31° 55′ N 130° 50′ E), where both species occur sympatrically, between April 26 and July 22, 2003, and between May 6 and July 17, 2004. They were classified into four developmental stages: (A) emerging (the aboveground organs becoming visible through the leaf litter), (B) flowering (anthers and stigma becoming visible from the perianth tube), (C) wilting (with blackened tepals), and (D) fruiting (ovary becoming large and protruding from the dried out tepals). These stages were counted manually while walking along a fixed route of ca. 500 m, at intervals of approximately 3 weeks.

### High-throughput plant phylogenetic analysis

A phylogenetic tree of *Monotropastrum* plants was constructed based on MIG-seq, which encompasses microsatellite-associated reduced-representation DNA sequencing with restriction site-associated DNA sequencing (RAD-seq) (Suyama and Matsuki 2015). After extracting the genomic DNA from the silica-dried samples using the cetyltrimethylammonium bromide (CTAB) method, we prepared an MIG-seq library, as per Suetsugu et al. (2021b) and Suyama et al. (2022). This included 32 *M. kirishimense* samples from seven populations, 19 *M. humile* samples from eight populations, including two *M. humile* f. *roseum* individuals, and one *Monotropastrum* sp. 1 sample (Table S1). The library was sequenced using an Illumina MiSeq Sequencer (Illumina, San Diego, CA, USA) with a MiSeq Reagent Kit v. 3 (150 cycle, Illumina). The raw MIG-seq data were deposited in the DDBJ Sequence Read Archive (DRA, accession number DRA014598).

After removing the primer regions and low-quality sequencing reads (Suetsugu et al. 2021b), 7,014,511 reads (137,539 ± 7234 reads per sample) were obtained from the original 7,804,056 raw reads (153,021 ± 7863 per sample). The Stacks v. 2.60 pipeline was used for de novo single-nucleotide polymorphism (SNP) discovery (Rochette et al. 2019). The following parameters were used: minimum depth of coverage required to create a stack (*m*) = 3, maximum distance allowed between the stacks (*M*) = 2, number of mismatches allowed between the sample loci when building the catalog (*n*) = 2. Only SNPs retained by 26 or more samples were extracted, and SNPs with high heterozygosity (Ho ≥ 0.6) were removed. Moreover, SNP sites with fewer than three minor alleles were filtered out. Finally, 1000 SNPs from 543 loci were provided for the subsequent analyses. SNP-based maximum likelihood (ML) phylogeny was inferred using RAxML v. 8.2.10 (Stamatakis 2014), with a GTR substitution model with Lewis’ ascertainment bias correction and 1000 bootstrap replicates.

### Molecular analysis of the mycorrhizal fungi

Genomic DNA was extracted from the root tips of 10 *Monotropastrum kirishimense* plants from four populations (Table S1), and 23 *M. humile* plants (including two *M. humile* f. *roseum* plants) from five populations, using CTAB methods. We amplified the ITS region of the mycorrhizal fungi using the primer set ITS86F/ITS4 (Waud et al. 2014) fused with 3–6-mer Ns and with the Illumina forward/reverse sequencing primer. To add the Illumina sequencing adapters, supplemental PCR was also performed as described in Suetsugu et al. (2021a, b). Equal volumes of each PCR amplicon were pooled and purified using the AMPure XP Kit (Beckman Coulter, CA, USA). The sequencing libraries were processed in an Illumina MiSeq sequencer, with the MiSeq Reagent Micro Kit v. 2 (300 cycles, Illumina, USA). The sequence data were deposited in the DRA (accession number DRA013047).

After sequencing, we performed bioinformatic analysis using Claident v. 0.2.2019.05.10 (Tanabe and Toju 2013), as described in Suetsugu and Matsubayashi (2021). Erroneous sequence reads were removed based on the CD-HIT-OTU method (Li et al. 2012), using the clcleanseqv command in Claident. The remaining sequencing reads were clustered into operational taxonomic units (OTUs) at a 97% threshold similarity, using VSEARCH v. 2.8.0 (Rognes et al. 2016). The OTUs were subjected to de novo and reference-based chimera removal, based on the UCHIME algorithm (Nilsson et al. 2019). The OTU taxonomic assignment was performed based on the query-centric auto-k-nearest-neighbor (QCauto) and the lowest common ancestor (LCA) algorithms (Tanabe and Toju 2013). The functional guild for each fungal OTU was estimated using the FUNGuild database (Nguyen et al. 2016). In subsequent analyses, we used the taxa designated as ectomycorrhizal fungi by FUNGuild, because all monotropoid species obtain their carbohydrates from mycorrhizal fungi that link them to the surrounding trees, on which the fungi form ectomycorrhizae (Bidartondo and Bruns 2001, 2002; Matsuda et al. 2011; Yokoyama et al. 2005).

Because all the *Monotropastrum kirishimense* and *M. humile* plants were predominantly colonized by OTUs assigned to *Russula*, we downloaded several *Russula* sequences closely related to the OTUs detected here, based on BLAST searches, from the International Nucleotide Sequence Database Collaboration (INSDC) database. The sequences obtained were aligned using ClustalW in MEGA X (Kumar et al. 2018). The aligned sequences were then used to reconstruct phylogenetic relationships using MEGA X (Kumar et al. 2018) with ML analysis, with a GTR + I + G model and 1000 bootstrap replicates (*lnL* =  − 3108.04). Our infrageneric classification of *Russula* follows Shimono et al. (2004), Looney et al. (2018), Wang et al. (2019), and Buyck et al. (2020).

## Results

### Morphological characters

**Fig. 1:**
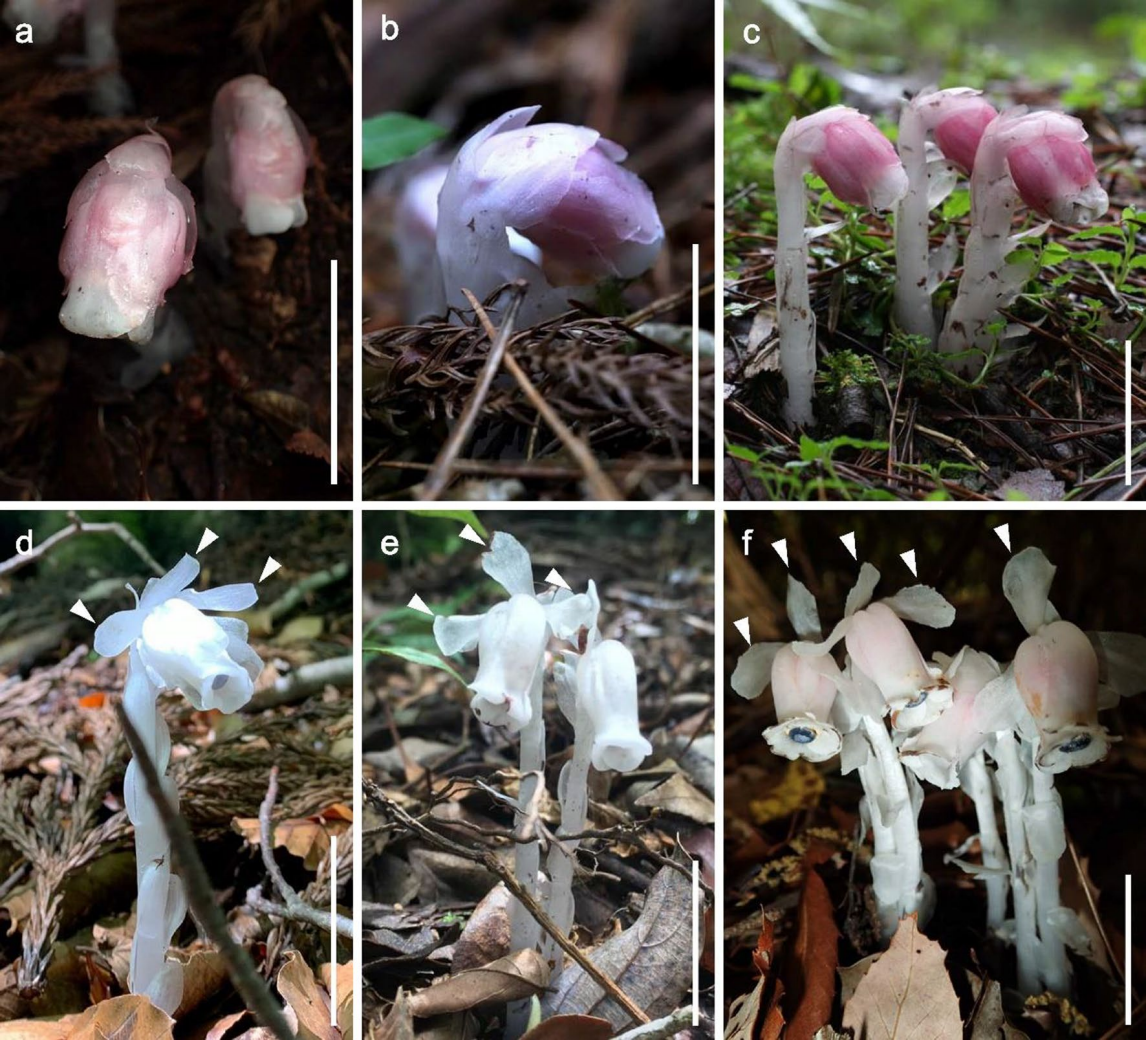
Morphological comparison of the aboveground parts of *Monotropastrum kirishimense* and *M. humile. Monotropastrum kirishimense* in **a** Fujieda-shi, Shizuoka Pref., **b** Ena-shi, Gifu Pref., and **c** Kirishima-shi, Kagoshima Pref. *Monotropastrum humile* in **d** Waga-gun, Iwate Pref. and **e** Tarumizu-shi, Kagoshima Pref., and **f** *M. humile* f. *roseum* in Sakyo-ku, Kyoto Pref. Arrowheads indicate spreading sepals. Scale bars: 3 cm. Photographed by Masayuki Sato (**a**), Katsumi Iwahori (**b**), Shuichi Kurogi (**c**), Shin Terui (**d**), Kazushige Uemori (**e**), and Kenji Suetsugu (**f**)

**Fig. 2:**
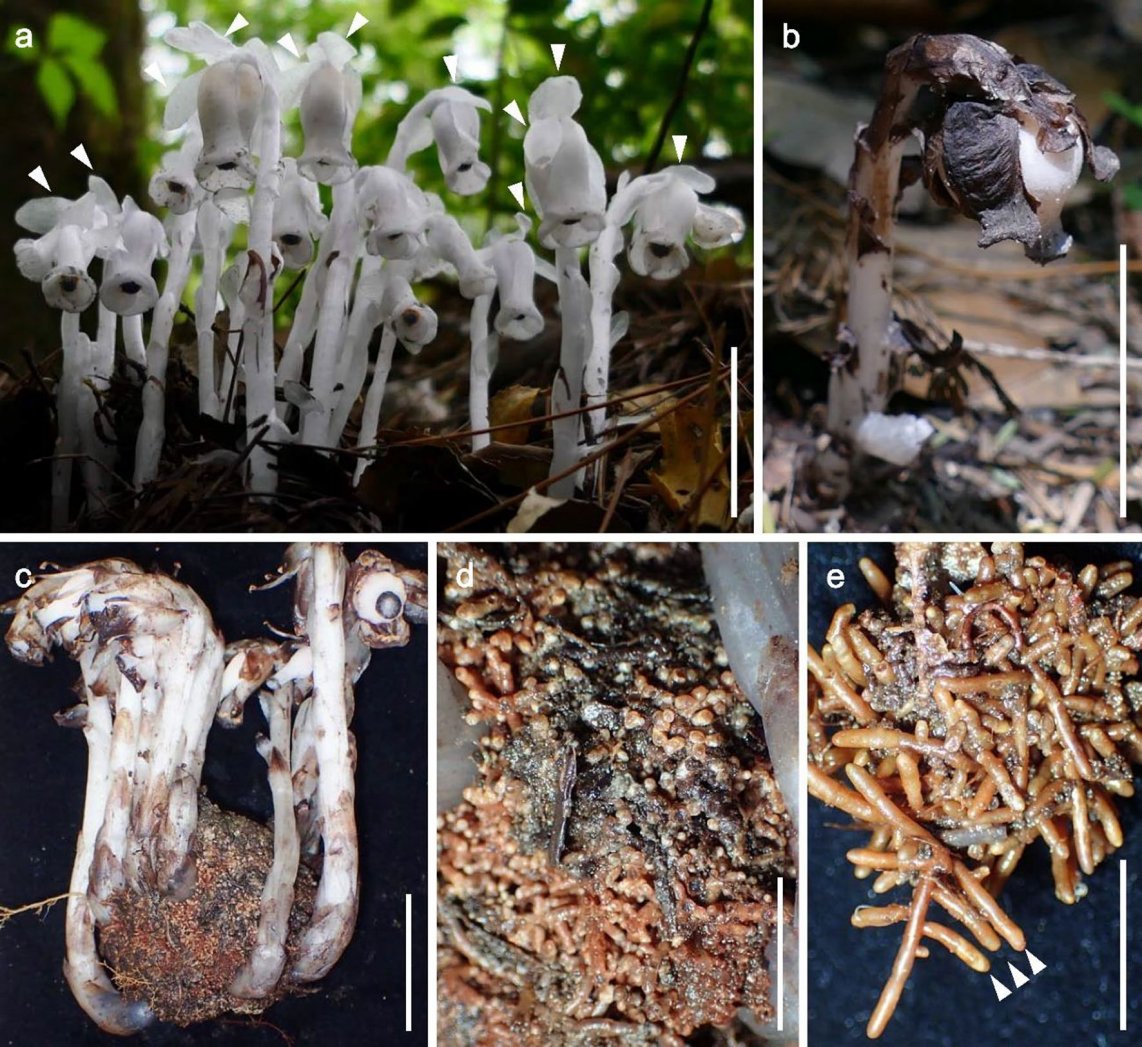
*Monotropastrum humile* and its monotropoid association found at the *M. kirishimense* type locality (on June 25, 2019, except for the flowering plants). **a** Flowering plants (on May 17, 2019). Arrowheads indicate spreading sepals. **b** Fruiting plant. **c** Fruiting scapes with root ball. **d**, **e** Magnification of the root ball. Root tips and branching are easily recognizable. Arrowheads indicate root tip apices. Scale bars: 3 cm (**a**–**c**), 1 cm (**d**), and 5 mm (**e**). Photographed by Hideo Shimada (**a**) and Kenji Suetsugu (**b**–**e**)

**Fig. 3:**
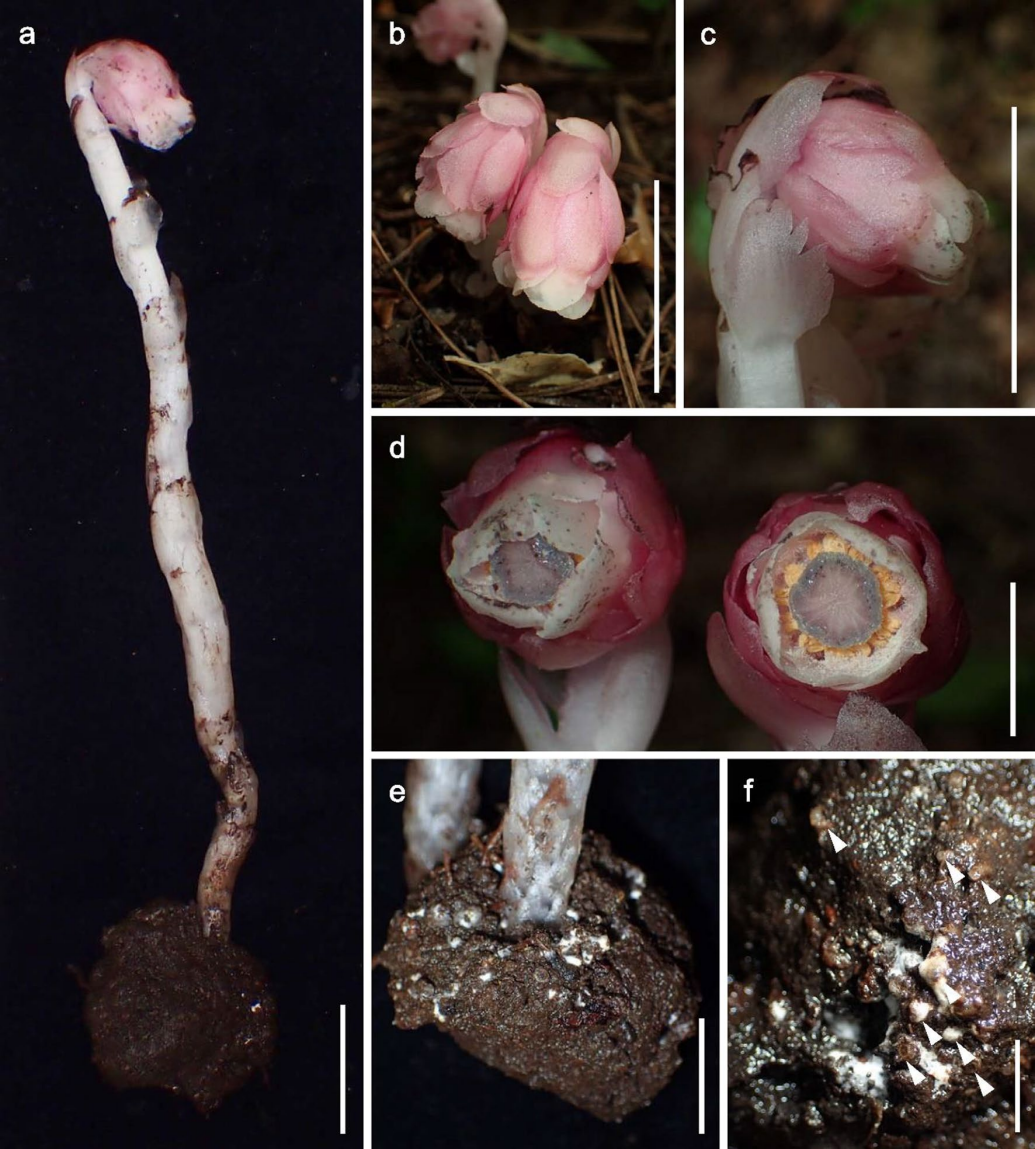
*Monotropastrum kirishimense* and its monotropoid association (holotype). **a** Flowering scape with root ball. **b**, **c** Flowering plants. **d** Flowers, top view. **e**, **f** Magnification of the root ball. Root tips are not apparent, but white fungal hyphae are visible. Arrowheads indicate the root tip apices. Scale bars: 3 cm (**a**–‍**c**), 1 cm (**d**, **e**), and 5 mm (**f**). Photographed by Kenji Suetsugu

**Fig. 4:**
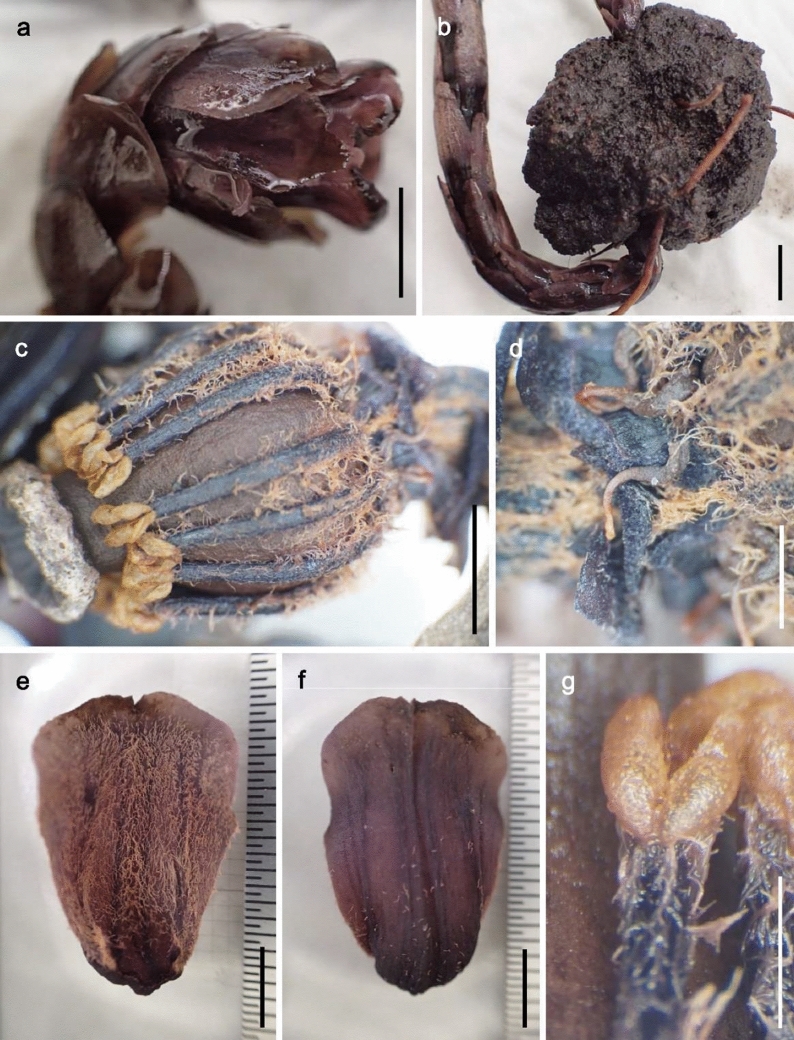
*Monotropastrum kirishimense* (holotype). **a** Flower. **b** Root ball with the interpenetrating *Pinus densiflora* root system. **c** Flower with perianth removed. **d** Floral discs with the basal part of the filaments and protrusions from the floral disc. **e** Petal, adaxial view. **f** Petal, abaxial view. **g** Anther. Scale bars: 1 cm (**a**, **b**), 5 mm (**c**, **e**, **f**), and 2 mm (**d**, **g**). Photographed by Kumi Hamasaki

**Fig. 5:**
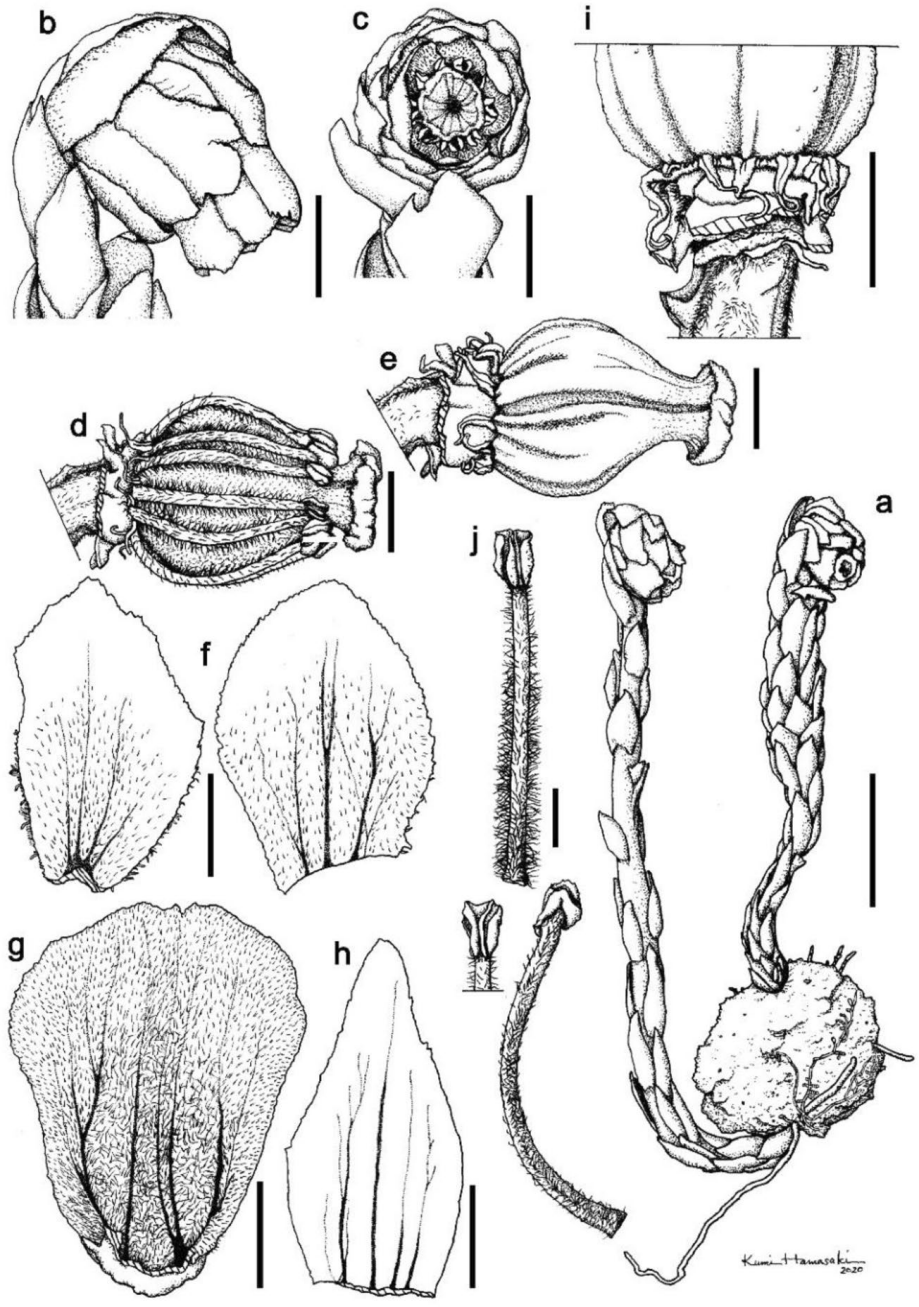
*Monotropastrum kirishimense* (drawn from the holotype). **a** Flowering scapes with root ball, with the interpenetrating *Pinus densiflora* root system. **b** Flower, side view. **c** Flower, top view. **d** Flower after the removal of perianth. **e** Flower after the removal of perianth and filaments. **f** Sepals, adaxial view. **g** Petal, adaxial view. **h** Scale leaf, adaxial view. **i** Floral discs and basal part of ovary. **j** Filaments. Scale bars: 3 cm (**a**), 1 cm (**b**, **c**), 5 mm (**d**–**i**), and 2 mm (**j**). Drawn by Kumi Hamasaki

Review and analysis of herbarium specimens, protologues, and living plants revealed few morphological characters that consistently differed between *Monotropastrum kirishimense* and *M. humile* (Figs. 1, 2, 3, 4, 5). Although the shape of the floral disc is recognized as a diagnostic character between *M. humile* and its closely related species (Tsukaya et al. 2008), the floral disc of *M. kirishimense* has thin protrusions elongated to bend backward (Fig. 4d), similar to that of *M. humile* (Tsukaya et al. 2008).

However, *Monotropastrum kirishimense* can be distinguished from *M. humile* by its rosy pink tepals and several other features. First, the flowers of *M. kirishimense* usually bear 4–9 (up to 11) generally elliptic sepals that are constantly appressed to the petals throughout its flowering period, while the flowers of *M. humile* usually bear 2–3 (up to 5) generally oblong sepals that are usually spreading during peak anthesis (Figs. 1, 2, 3, 4, 5). Second, *M. kirishimense* flowers and ovaries are more rounded, with a lower aspect ratio than those of *M. humile*. Although the *M. humile* ovary also expands and becomes rounded after pollination, the flowers and ovaries of *M. kirishimense* are stout even before pollination (Figs. 1, 2, 3, 4, 5). Third, *M. kirishimense* is always shorter above ground (typically < 5 cm, vs. > 5 cm in *M. humile*; Figs. 2a, 3b), whereas the underground stalk is longer in *M. kirishimense* (typically > 10 cm, vs. < 5 cm in *M, humile*; Figs. 2c, 3a). More importantly, as suggested by Imamura and Kurogi (2003), the root ball morphology differs completely between these two species. The *M. kirishimense* root ball is obscure and unified with the surrounding soil, with little protrusion of root tips, and a white mantle indicating mycorrhizal formation (Figs. 3f, 4b, 5a). Meanwhile, that of *M. humile* can easily be distinguished from the soil and has relatively distinct root tips (Fig. 2e).

Although *Monotropastrum humile* f. *roseum* is also distinguished from *M. humile* f. *humile* by its red flowers, Honda (1957) did not describe its characteristics other than floral coloration. Its holotype comprises only the aboveground parts. Nonetheless, due to the spreading oblong-elliptic to obovate-elliptic sepals, we can conclude that *M. kirishimense* is not conspecific with *M. humile* f. *roseum.* Additional sampling of *M. humile* f. *roseum* indicates that *M. humile* f. *roseum* cannot be distinguished from *M. humile* f. *humile*, other than in coloration. Additional sampling has also shown that *M. humile* f. *roseum* has a reddish ovary (Fig. 1f), and that *M. kirishimense* has rosy pink tapels (Fig. 1a–c).

### Flowering phenology

**Fig. 6:**
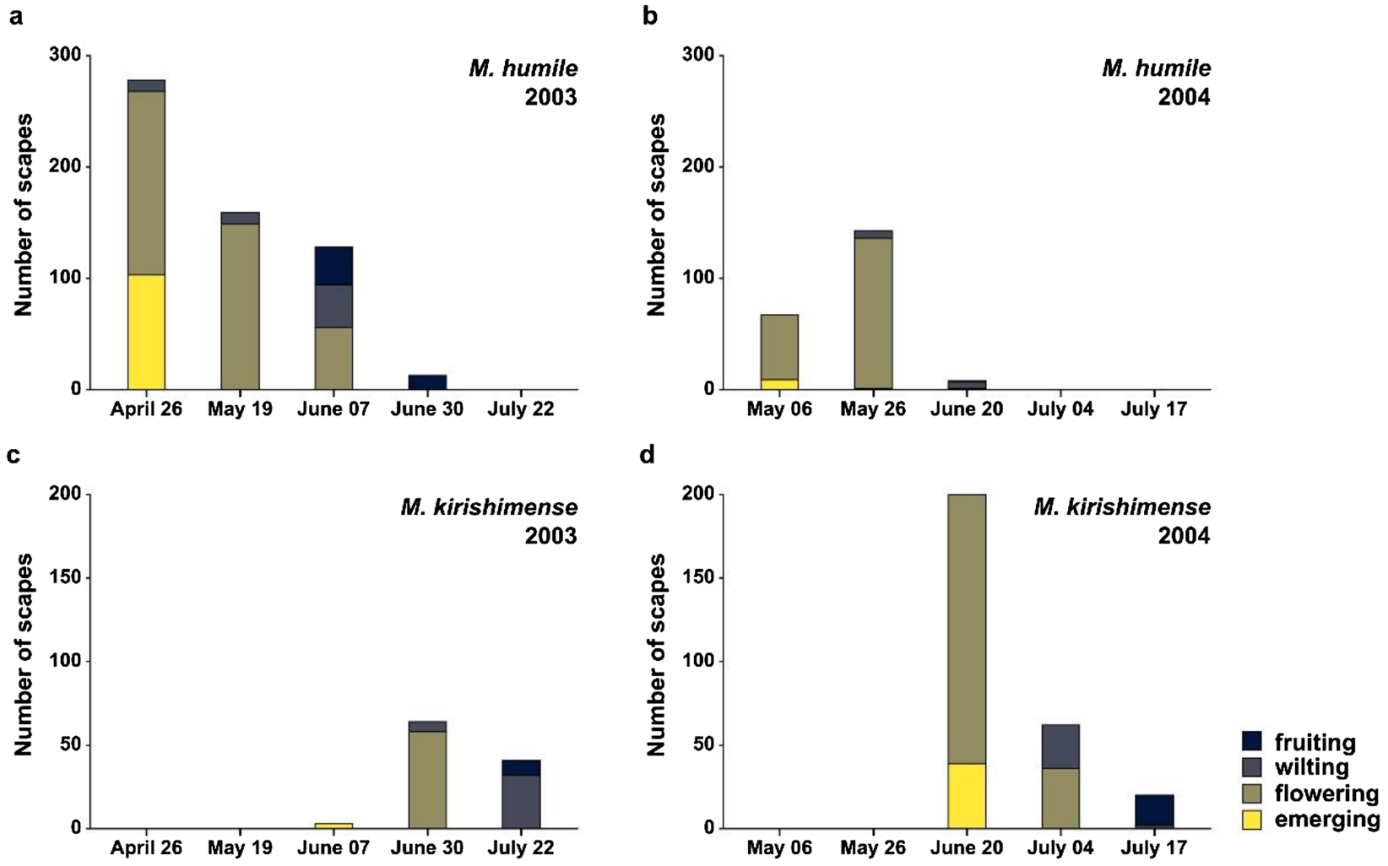
Flowering phenology of *Monotropastrum kirishimense* (**a**, **b**) and *M. humile* (**c**, **d**) in the Onami population (31°55’N 130°50’E), where both species occur sympatrically

*Monotropastrum humile* flowers mature much earlier than those of *M. kirishimense* (Fig. 6)*.* On 19 May 2003, all the *M. humile* plants were in bloom or had already begun to wilt, whereas no *M. kirishimense* plants were visible. On 30 June 2003, many *M. humile* plants had already disappeared, with the few remaining individuals all being at the fruiting stage. Meanwhile, most of the *M. kirishimense* individuals were flowering. These *M. humile* and *M. kirishimense* plants reached almost equivalent flowering stages on 19 May 2003 and 30 June 2003, respectively, indicating that *M. humile* flowers ca. 40 days before *M. kirishimense.*

### Plant phylogeny

**Fig. 7:**
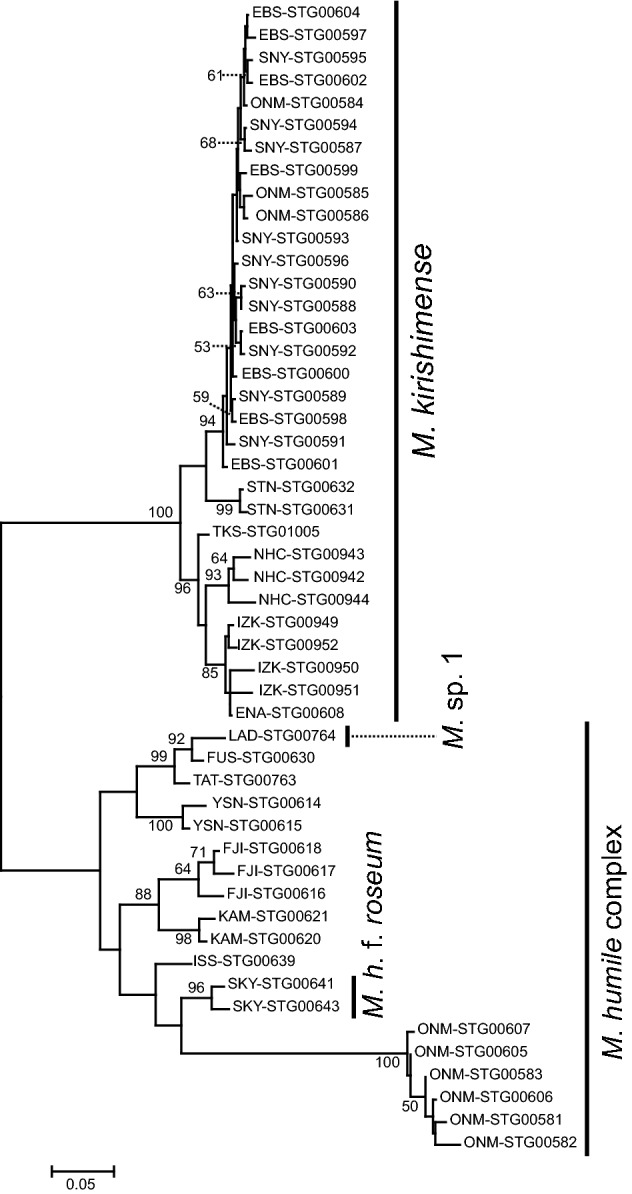
Phylogenetic tree of *Monotropastrum kirishimense* and *M. humile* reconstructed from MIG-seq data. Nodes supported by bootstrap values < 50% are not shown. Branch length represents the average number of substitutions per site

The *Monotropastrum* ML phylogenetic tree separated *M. kirishimense* from the remaining taxa, and the monophyly of each clade was highly supported (a 100% bootstrap value; Fig. 7). Although *Monotropastrum* sp. 1 is not morphologically identical to *M. humile*, because of its glabrous flower organs and broad, somewhat ridged fruits, it was embedded within a clade comprising the remaining *M. humile.* The genetic differentiation between *Monotropastrum* sp. 1 and *M. humile* was not as large as that between *M. kirishimense* and *M. humile.* Therefore, it might be appropriate to consider it an intraspecific variant of *M. humile.* However, considering that *Monotropastrum* sp. 1 is distinguished from *M. humile* by many morphological traits, further investigation would help to determine whether there are cryptic *Monotropastrum* species other than *M. kirishimense* within the species complex.

### Fungal community

**Fig. 8:**
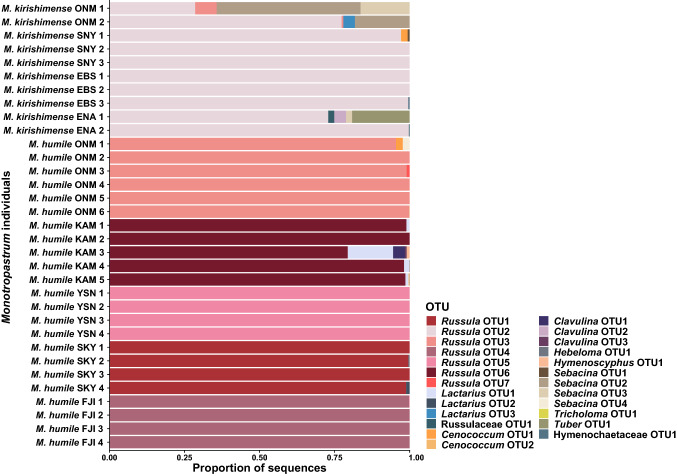
Relative abundance of ectomycorrhizal communities associated with *Monotropastrum kirishimense* and *M. humile*, at the operational taxonomic unit (OTU) level

Of the 93 fungal operational taxonomic units (OTUs) (204,485 sequencing reads) retrieved using the fungal ITS primer set, 25 OTUs (185,571 reads; 90.8% of all reads) were considered putative ectomycorrhizal fungi (Table S2). Most of the fungal ITS sequences of the *Monotropastrum kirishimense* and *M. humile* mycobionts had high DNA-sequence homology with *Russula* species (seven OTUs, 182,693 reads; 89.3% of all reads, Fig. 8) (Table S2)*.* All the *M. kirishimense* plants were predominantly colonized by the same *Russula* OTU (*Russula* OTU2; 32,308 sequencing reads; 84.7% of all reads). This dominant association between *M. kirishimense* and a single *Russula* OTU among multiple populations more than 700 km apart provides strong evidence that *M. kirishimense* exhibits specialized interactions with this OTU (Table S2, Fig. 8). Only one other *Russula* OTU (*Russula* OTU3; 12 reads) was detected, in two samples; given its extremely low number of sequencing reads, this is likely an opportunistic fungus with no fundamental role for *M. kirishimense*. Hence, we conclude that *M. kirishimense* is primarily specialized on *Russula* OTU2. For each of the *M. humile* individuals, one of five *Russula* OTUs was the predominant colonizer (150,373 reads; 90.4% of all reads; Table S2)*.* However, *Russula* OTU2, dominant in *M. kirishimense*, was not detected in any of the *M. humile* individuals, even in the sympatric Onami population.

**Fig. 9:**
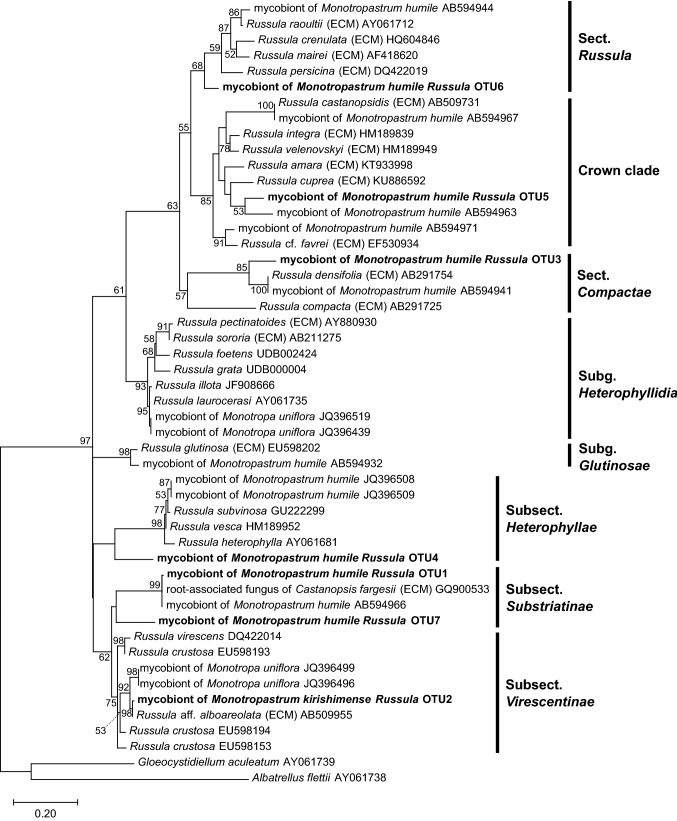
Phylogenetic tree of partial internal transcribed spacer rDNA sequences from mycorrhizal fungi of the *Monotropastrum kirishimense* and *M. humile* plants investigated in this study (bold type) and from the International Nucleotide Sequence Database Collaboration (INSDC) database. Nodes supported by bootstrap values < 50% are not shown

Our fungal ITS sequence ML phylogenetic analysis has shown that the *Monotropastrum kirishimense* mycobionts were clustered nearest to sequences of *Russula* aff. *alboareolata* (AB509955), belonging to the subsection *Virescentinae* (Fig. 9)*.* This subsection has not been reported as a mycobiont of the 93 M*. humile* individuals collected in Japan, Taiwan, and China across 15 populations (Bidartondo and Bruns 2001; Matsuda et al. 2011; Min et al. 2012; Yokoyama et al. 2005)*.* In contrast, all the *Russula* OTUs associated with the *M. humile* individuals investigated in the present study formed a clade with mycobionts previously detected in *M. humile* (Fig. 9).

## Discussion

Our morphological investigation has indicated that *Monotropastrum kirishimense* can be distinguished from *M. humile* by its rosy pink tepals, more numerous elliptic sepals constantly appressed to the petals throughout its flowering period, and obscure root balls unified with the surrounding soil, with little protrusion of the root tips. Our MIG-seq-based phylogenetic tree has also shown that *M. kirishimense* and the other *M. humile* species complex can be separated into two monophyletic clades, with a 100% bootstrap value. We have therefore determined that *M. kirishimense* should be treated as an independent species, based on its morphological and phylogenetic distinctness.

Floral trait differentiation between taxa plays a key role in reducing interspecific pollen transfer, either through phenological isolation (a pre-mating barrier caused by differences in flowering time) or floral isolation (a pre-mating barrier caused by differences in morphological, visual, or olfactory traits) (Chapurlat et al. 2020). Given their overall similarity in floral features, the two species are unlikely to experience pollinator-mediated isolation. Indeed, we have observed that *Monotropastrum kirishimense* and *M. humile* are mainly pollinated by the bumblebee *Bombus diversus* (K. Suetsugu, unpublished data)*.* Reproductive isolation between *M. kirishimense* and *M. humile* is also unrelated to the spatial separation of these species, given that they grow adjacent to each other.

Phenological isolation between plant taxa has substantial potential to lead to reproductive isolation. *Monotropastrum humile* flowers more than a month before *M. kirishimense*, with only a brief period (if any) of overlap at the end of *M. humile* flowering. This divergence in flowering time could be directly selected as a reinforcement mechanism to reduce hybridization before complete speciation (Osborne et al. 2020). Therefore, speciation between *M. kirishimense* and *M. humile* may be reinforced by differences in the timing of floral maturation. However, it is also possible that flowering-time divergence could be selected after speciation is complete, as a mechanism to avoid wasting reproductive effort on unfit hybrids (Hopkins 2013). Reproductive asynchrony in flowering time can reduce heterospecific pollen deposition, helping to ensure conspecific mating (Lowry et al. 2008).

Metabarcoding-based community profiling revealed that *Monotropastrum kirishimense* and *M. humile* are predominantly associated with different *Russula* lineages. *Monotropastrum kirishimense* is consistently associated with *Russula* OTU2 (in subsection *Virescentinae*), even in the Onami population, where *M. humile*, associated with different OTUs, grows sympatrically within a few meters. Therefore, we can conclude that their genetic characteristics drive the differences in mycorrhizal interaction between the two species. For *M. kirishimense*, the association with *Russula* OTU2 encompasses four sampling localities spanning the geographic distribution of the species. Despite extensive studies on its mycorrhizal communities based on 113 individuals collected in Japan, Taiwan, and China across 20 populations (Bidartondo and Bruns 2001; Matsuda et al. 2011; Min et al. 2012; Yokoyama et al. 2005; present study), the subsection *Virescentinae* has never been reported as a mycobiont of *M. humile*.

In contrast, all the *Russula* OTUs associated with *M. humile* individuals collected from all the five populations in our studies were closely related or identical to OTUs previously reported as mycobionts of *M. humile* (Bidartondo and Bruns 2001; Matsuda et al. 2011; Min et al. 2012; Yokoyama et al. 2005). Consequently, we conclude that the two species differ in mycorrhizal specificity, and that *M. kirishimense* has a specialized association with *Russula* OTU2, although more extensive assessments may reveal that some *M. humile* individuals are associated with *Russula* OTU2.

Although *M. kirishimense* is widely distributed in Japan, it is specialized on *Russula* OTU2, and is much rarer locally than *M. humile*, which is associated with diverse members of the Russulaceae. Although the high host specificity of *M. kirishimense* may contribute to its rarity, the ecology of its fungal OTU, including its preferred habitat, soil requirements, or fidelity to a specific ectomycorrhizal host tree, remains unexplored. Notably, *M. kirishimense* occurs only in coniferous forests (dominated primarily by *Pinus densiflora*), while *M. humile* commonly occurs in not only coniferous forests, but also other ectomycorrhizal forests, such as fagaceous forests. *Russula* OTU2 may be preferentially associated with *P. densiflora.* Further studies are needed to investigate the distribution patterns and abundance of *Russula* species associated with *M. kirishimense* and *M. humile*.

Our findings imply that the distinct mycorrhizal communities play a crucial role in the niche partitioning and coexistence of *M. kirishimense* and *M. humile*. Because (i) classical theoretical ecology predicts that two species competing for the same resources cannot stably coexist (Gause 1934) and (ii) mycoheterotrophic plants depend on mycorrhizal fungi for their carbon demands (Merckx 2013), divergent mycorrhizal associations can play a vital role in reducing resource competition. Previous studies have also showed that sympatric (at least initially) mycoheterotrophic plants often have distinct mycorrhizal communities and display strong spatial segregation, even if they share some fungal OTUs (Bidartondo and Bruns 2005; Jacquemyn et al. 2014; McCormick and Jacquemyn 2014; Taylor and Bruns 1999). Our findings may therefore indicate that niche differentiation via segregation of mycorrhizal fungi represents an important mechanism contributing to sympatry. Given that vertical depth partitioning among closely related ectomycorrhizal fungi is a common phenomenon (Mujic et al. 2016; Taylor et al. 2014), the different root depths of *M. kirishimense* and *M. humile* may be an adaptation to effectively exploit vertically separated fungal partners.

Furthermore, speciation between *Monotropastrum kirishimense* and *M. humile* may be partially due to resource partitioning, with specialization on different fungal hosts leading to reproductive isolation (see also Barrett and Freudenstein 2011; Barrett et al. 2022). Speciation via host shift is one of the most plausible modes of ecological speciation (Calcagno et al. 2007; Fry 2003). This scenario begins with the formation of host races comprising host-affiliated, genetically differentiated groups within the parental species (Drès and Mallet 2002; Jacquemyn et al. 2018). Disruptive selection on host-specificity, indicated by trade-offs in performance between hosts, can lead to further specialization, and promote the formation of two daughter species (Jacquemyn et al. 2018; Rundle and Nosil 2005).

Although little is known about the genetic basis of mycoheterotroph–mycorrhizal associations, the high mycorrhizal specificity observed in many mycoheterotrophs is thought to be the result of physiological fine-tuning to adapt to particular fungi (Hynson and Bruns 2009). Breakdown of coadapted gene complexes controlling host specificity may, therefore, be responsible for postzygotic isolation, in the form of reduced hybrid fitness. Hybridization between ecotypes within a single mycoheterotrophic species with different host specificity can considerably reduce progeny fitness due to a lower probability of mycoheterotrophic growth (Jacquemyn et al. 2016, 2018). Consequently, differences in mycorrhizal communities have been suggested to contribute to reproductive isolation among mycoheterotrophic plants (Barrett and Freudenstein 2011; Barrett et al. 2022; Jacquemyn et al. 2018). Future investigations, including artificial interspecific cross-pollination experiments and in-situ seed baiting, are required to determine whether mycorrhizal associations can prevent hybrid seeds from establishing successful seedlings, thus acting as a post-mating barrier in these two *Monotropastrum* species. We also note that genotypically distinct *M. humile* individuals in the different populations tended to be predominantly colonized by different *Russula* OTUs, highlighting potential race formation within *M. humile.* However, it is impossible to exclude the possibility that the local availability of *Russula* species is the primary determinant, because there was no geographic mixing of different genotypes. More extensive sampling across a much broader geographic range would facilitate a more robust understanding of the evolutionary dynamics of mycorrhizal specificity within *M. humile.*

In summary, we have shown that *Monotropastrum kirishimense* is distinct from *M. humile* based on morphology, flowering phenology, and the molecular identity of itself and its fungal partners. Phenological differences (a pre-mating reproductive barrier) and distinct mycorrhizal specificity (a post-mating reproductive barrier) are likely to contribute to the ongoing sympatry of *M. kirishimense* and *M. humile*. Mycoheterotrophic plants are often susceptible to environmental destruction, because they are highly dependent on the fungi and the trees that sustain them (Suetsugu et al. 2020). Therefore, many members of the Monotropoideae are restricted to old-growth forests and are now in danger of extinction (Min et al. 2012). The rare and previously unrecognized *M. kirishimense* can now receive conservation recognition for the first time. This study highlights the importance of integrative taxonomy to avoid under-assessing biodiversity.

### Taxonomic treatment

***Monotropastrum kirishimense ***Suetsugu, *sp. nov.* (Figs. 1a–c, 3, 4, 5)

**Type.** JAPAN. Kagoshima Pref, Kirishima-shi, Makizono-cho, Ohnami-ike, 25 June 2019, *Kenji Suetsugu KS424* (holotype: KYO!, dried plant on an herbarium sheet and liquid-preserved material in a bottle labeled as the same specimen; isotypes: TI!, TNS!, dried plant on an herbarium sheet).

**Diagnosis.** *Monotropastrum kirishimense* is similar to *M. humile* but differs in its rosy pink tepals, more numerous (4–11) elliptic sepals constantly appressed to the petals throughout its flowering period, and obscure root balls unified with the surrounding soil, with little protrusion of the root tips.

Terrestrial, mycoheterotrophic herb. Root ball unified with the surrounding soil, with little protrusion of the root tips, 4.7–6.3 cm in diam; roots 0.7–0.9 mm in diam. Stems erect, 8.5–20 cm long, 3.8–7.8 mm in diam. below flower, arising in nodding position from root ball; uniflorous. Scale leaves on upper stem narrowly ovate, 16–21 mm long, 6–10 mm wide, entire to erose, apex acute to rounded, glabrous. Scale leaves at the base of stem shorter and more densely crowded on axis, glabrous. Flower campanulate, solitary, nodding at anthesis, 15.3–25 mm long, 11.1–13.9 mm wide at the middle, 10.1–17.0 mm at apex. Sepals 4–9(–11), rosy pink, elliptic, 14.2–19.8 mm long, 8.0–12.6 mm wide, appressed to petals, slightly erose, abaxially glabrous or slightly pubescent, adaxially pubescent. Petals (3–)4–5, rosy pink, obovate-oblong to cuneate-oblong, 16.8–20.3 mm long, 11.1–14.8 mm wide, entire, abaxially glabrous or slightly pubescent, adaxially densely pubescent, base broadly saccate, apex dilated. Stamens 10–14; filaments 10.8–13 mm long, pubescent; anthers yellow, horizontally reniform, 1.6–2.2 mm long, 0.9–1.6 mm wide, with a single terminal slit across connate sacs. Pollen grains monad 23–30 μm in diam., commonly triporate, pores protruding, fine verrucate–rugulate. Style 2.5–3.2 mm long, merging imperceptibly with apex of the ovary. Stigma funnel-form, blue on margin, ca. 1.5 mm long, 5–6 mm in diam. Ovary ovoid, unilocular, without distinct ridges, 9.5–15.8 mm long, 9–11.2 mm wide, glabrous; parietal placentae 10–14. Fruit white, erect to nodding, ovoid-globose, abruptly narrowed to style, 10.1–18.7 mm long, 10.6–23.5 mm wide, interior; seeds numerous, embedded within fleshy pulp. Seeds ovoid, ca. 0.4 mm long, ca. 0.2 mm wide; testa not prolonged, minutely reticulate.

**Additional specimens examined (paratype).** JAPAN. **Kyushu District**—Kagoshima Pref.: Kirishima-shi, Mt. Eboshi, 25 June 2019, *Kenji Suetsugu KS426* (KYO); Kirishima-shi, Mt. Eboshi, 26 June 2010, *Kenji Suetsugu Mk1* (KYO); Kirishima-shi, Makizono-cho, Ohnami-ike, 26 June 2010, *Kenji Suetsugu Mk2* (KYO); Kirishima-shi, Makizono-cho, Ohnami-ike, 18 June 2010, *Shuichi Kurogi MZ45233* (MZ); Kirishima-shi, Makizono-cho, Shinyu, 25 June 2019, *Kenji Suetsugu KS427* (KYO); Tarumizu-shi, Onogaradake, 26 June 2022, *Hiromitsu Sakota et al. KAG181002* (KAG). Miyazaki Pref.: Ebino-shi, Obeno, 29 June 2014, *Masami Saito et al. MZ40210* (MZ); Ebino-shi, Rokkannon Mike, 18 June 2002, *Shuichi Kurogi MZ45234* (MZ). **Kinki District**—Wakayama Pref.: Tanabe-shi, Nakahechi-cho, 28 June 2020, *Tomoaki Ohe KS709* (KYO). Osaka Pref.: Izumisano-shi, Mt. Takashiro, 19 June 2021, *Tetsuro Ikeda M76-1* (KYO); Kaizuka-shi, Mt. Izumi Katsuragi, 27 June 2020, *Tetsuro Ikeda KS708* (KYO). **Chubu District**—Gifu Pref.: Ena-shi, Nakanoho-cho, 7 July 2017, *Katsumi Iwahori M1* (KYO); Ena-shi, Nakanoho-cho, 18 July 2018, *Katsumi Iwahori M11* (KYO); Ena-shi, Nakanoho-cho, 27 June 2020, *Katsumi Iwahori KS706* (KYO); Ena-shi, Nakanoho-cho, 27 June 2020, *Katsumi Iwahori KS707* (KYO). Shizuoka Pref.: Fujieda-shi, Setonoya, 8 July 2017, *Masayuki Sato M10-1* (KYO); Fujieda-shi, Setonoya, 8 July 2017, *Masayuki Sato M10-2* (KYO); Fujieda-shi, Setonoya, 20 June 2013, *Kenji Suetsugu Mk3* (KYO); Fujieda-shi, Mt. Ryuso, 17 July 2017, *Norio Nishiguchi M2* (KYO); Fujieda-shi, Mt. Ryuso, 20 June 2012, *Kenji Suetsugu Mk4* (KYO).

**Japanese name.** Kirishima-gin-ryo-so

**Etymology.** The species is named after the type locality, Kirishima. To distinguish it from *Monotropastrum humile* f. *roseum* (beni-bana-gin-ryo-so, in Japanese) described by Honda (1957), we use Kirishima-gin-ryo-so as a Japanese name, after the type locality.

**Distribution.** Japan [Kyushu District (Kagoshima and Miyazaki), Kinki District (Wakayama and Osaka), and Chubu District (Gifu and Shizuoka)]. During intensive fieldwork and herbaria surveys, we identified several previously unknown populations of this taxon, previously considered endemic to the area around Kirishima, Kagoshima. It has now been recognized in Kyushu and Honshu. It is likely that *M. kirishimense* also occurs in Kochi, Shikoku, where field photographs of similar plants are shown on the website (https://hanasakiyama.web.fc2.com/yasou/sp/Itiyakusou_Benibanaginryousou.htm). Because mycoheterotrophic plants are easily overlooked in the wild because of their short flowering season and dwarf habit, *M. kirishimense* may be more widely distributed. In addition, *M. kirishimense* has probably been confused with the more well-known *M. humile* with similar morphology. Therefore, further surveys during the flowering season may reveal a broader distribution for *M. kirishimense.*

**Conservation status.** While we have found that *Monotropastrum kirishimense* is distributed in the Kyushu, Kinki, and Chubu Districts, *M. kirishimense* is much rarer than *M. humile.* The populations often harbor fewer than 20 individuals each; at the type locality, which sustains the largest number of individuals, the population comprises fewer than 50 plants. Therefore, we consider the conservation status to be endangered (EN) according to the IUCN criteria (IUCN 2019), under criterion D, in which the number of mature individuals is less than 250.

## Supplementary Information

Below is the link to the electronic supplementary material.

Supplementary file1 (PDF 1603 KB)

Supplementary file2 (XLSX 24 KB)

## Data Availability

MIG-seq and fungal community data are deposited in the DRA (DRA014598 and DRA013047, respectively).
