# Supplementary material for: Monotropastrum kirishimense (Ericaceae), a new mycoheterotrophic plant from Japan based on multifaceted evidence
Source: J Plant Res. 2022 Nov 29;136(1):3–18. doi: 10.1007/s10265-022-01422-8 (PMC9832082; doi:10.1007/s10265-022-01422-8)
Supplement: Supplementary file 1 — Supplementary file1 (PDF 1603 KB) [file 10265_2022_1422_MOESM1_ESM.pdf]

Electronic Supplementary Material

Monotropastrum kirishimense (Ericaceae), a new mycoheterotrophic plant from Japan based on multifaceted evidence

Kenji Suetsugu, Shun K. Hirota, Tian-Chuan Hsu, Shuichi Kurogi, Akio Imamura, Yoshihisa Suyama

Corresponding author: Kenji Suetsugu
Department of Biology, Graduate School of Science, Kobe University, Kobe 657-8501, Japan
Tel: +81-78-803-5713
Fax: +81-78-803-5713
Email: kenji.suetsugu@gmail.com

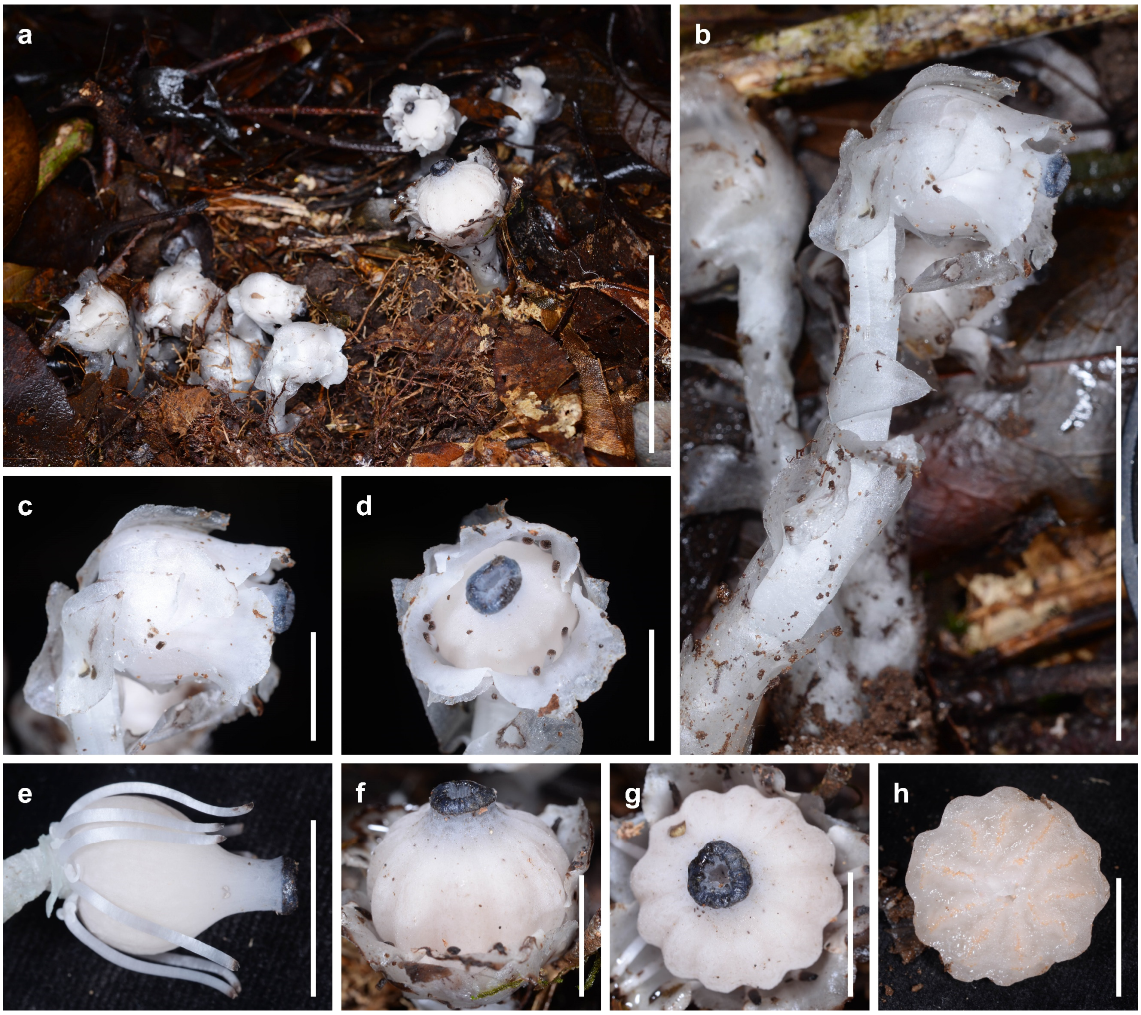

Fig. S1 *Monotropastrum* sp. 1 (*Hsu 10691*, TAIF). (a) Habit. (b) Flowering plants at the late flowering stage. (c) Flower at the late flowering stage., lateral view. (d) Flower at the late flowering stage, front view. (e) Flower with the petals and sepals removed, at the late flowering stage. (f) Fruiting ovary, lateral view. (g) Fruiting ovary, front view. (h) Fruiting ovary, sectional view. Scale bars: 5 cm (a–b), 1 cm (c–h). Photographed by Tian-Chuan Hsu.
